# Editorial: Nonalcoholic fatty liver disease: From pathogenesis to phytomedicine based on omics technology

**DOI:** 10.3389/fphar.2022.1062979

**Published:** 2022-10-21

**Authors:** Guang Ji, Guoxiang Xie, Tao Wu, Yunping Qiu

**Affiliations:** ^1^ Institute of Digestive Disease, Longhua Hospital, Shanghai University of Traditional Chinese Medicine, Shanghai, China; ^2^ University of Hawaii Cancer Center, Honolulu, HI, United States; ^3^ Institute of Interdisciplinary Integrative Medicine Research, Shanghai University of Traditional Chinese Medicine, Shanghai, China; ^4^ Albert Einstein College of Medicine, New York City, NY, United States

**Keywords:** NAFLD, pathogenesis, phytomedicine, omics, experimental pharmacology

Nonalcoholic fatty liver disease (NAFLD) including nonalcoholic fatty liver, nonalcoholic steatohepatitis (NASH), fibrosis, cirrhosis, and eventually hepatocellular carcinoma (HCC) has become the most common liver disease worldwide. No approved pharmacological treatments are available for NAFLD currently. Along with the development of analytical and phytochemical technologies, more and more phytochemicals with clear chemical structure have been identified with the potential to treat NAFLD.

This Research Topic aims to gather the novel discovery of molecular pathogenic mechanisms of NAFLD using multiomics, such as genomics, transcriptomics, proteomics and metabolomics. Simultaneously, novel findings on phytochemicals with clear chemical structure from traditional medical herbs, or natural plants, or novel natural compounds, active ingredients that can be used for NAFLD are collected.

Natural herbs are promising for their effect on ameliorating lipid metabolic disorders. Theabrownin (TB) and *Poria cocos* polysaccharide (PCP) have been reported to treat hyperlipidemia and diabetes. Wang et al. compared the effect of individual TB or PCP and the combination of TB and PCP (TB + PCP) on NAFLD phenotypes and the alteration of metabolism in the mice fed with high-fat diet. It was shown that TB, PCP, and TB + PCP reduced serum and hepatic lipid levels, among which TB + PCP was the most effective. Serum metabolomics profile and liver mRNA analyses revealed that the treatments alleviated the altered metabolic pathways including fatty acid metabolism, bile acid (BA) metabolism, and tricarboxylic acid cycle. They concluded that TB, PCP as well as the combination of TB and PCP could be potential therapeutic formula for NAFLD through promoting lipid utilization and inhibiting lipid synthesis and absorption.

Changes in BA are increasingly recognized as potential targets for NASH. Kaempferol has been demonstrated to have anti-inflammatory roles and regulate the disorder of lipid metabolism. In order to analyze BA profile in NASH mice, and determine the predictive biomarkers of kaempferol treatment, Lu et al. performed a serum targeted metabolomics and liver tissue RNA sequence from 6 normal control mice (NC group), 8 HFD-fed mice (HFD group), 8 kaempferol-treated with HFD-fed mice (HFD + KP group). They used targeted metabolomics based on ultra-performance liquid chromatography coupled to tandem mass spectrometry system to quantify serum and liver BAs, and RNA-sequence to detect liver differentially expressed genes related to BA metabolism. They reported that serum βMCA, CA, UDCA, 12-DHCA, ωMCA, CDCA, TωMCA, TDCA, THDCA, TCDCA and TUDCA, as well as liver 6,7-diketoLCA,12-DHCA and ωMCA may be potential biomarkers for kaempferol to improve NASH. HFD-induced NASH may be associated with the increase of CYP7A1 and the decrease of CYP8B1 leading to increased BA synthesis and the decrease of MRP3 leading to decreased BA synthesis, and kaempferol may improve NASH through increasing CYP27A1 and NTCP to enhance BA transport.

NASH is a clinical syndrome with pathological changes. Till now, there is lack of specific and effective treatments for NASH and NASH-driven HCC, and the detailed mechanisms of the progression of NASH to HCC are unclear. Therefore, it is urgent to understand the pathogenesis and progression of these diseases to identify new therapeutic approaches. Currently, an increasing number of researches are focusing on the utility of natural products in NASH, which is likely to be a promising prospect for NASH. Shao et al. reviewed the possible mechanisms of the pathogenesis and progression of NASH and NASH-derived HCC, and highlighted several natural products that have been shown to inhibit the conversion of NASH to HCC, as well as a lot of natural compounds with great potential to play a role in this process.

Better prevention and treatment strategies are required to improve the impact of NAFLD. Although liver biopsy is an effective tool for diagnosing NAFLD, it is invasive and difficult to perform for patients clinically. Therefore, it is very important to develop more efficient and noninvasive approaches for the early diagnosis of NAFLD. TCM can play a certain role in improving symptoms and protecting target organs in NAFLD, and its mechanism of action needs to be further studied. Metabolomics can provide useful clinically biomarkers that can be applied to NAFLD. Based on PubMed/MEDLINE and other databases, Shao et al. retrieved relevant literatures on NAFLD and TCM intervention using metabolomics technology in the past 5 years and summarized the specific metabolites associated with the development of NAFLD and the potential mechanism of Chinese medicine.

**FIGURE 1:**
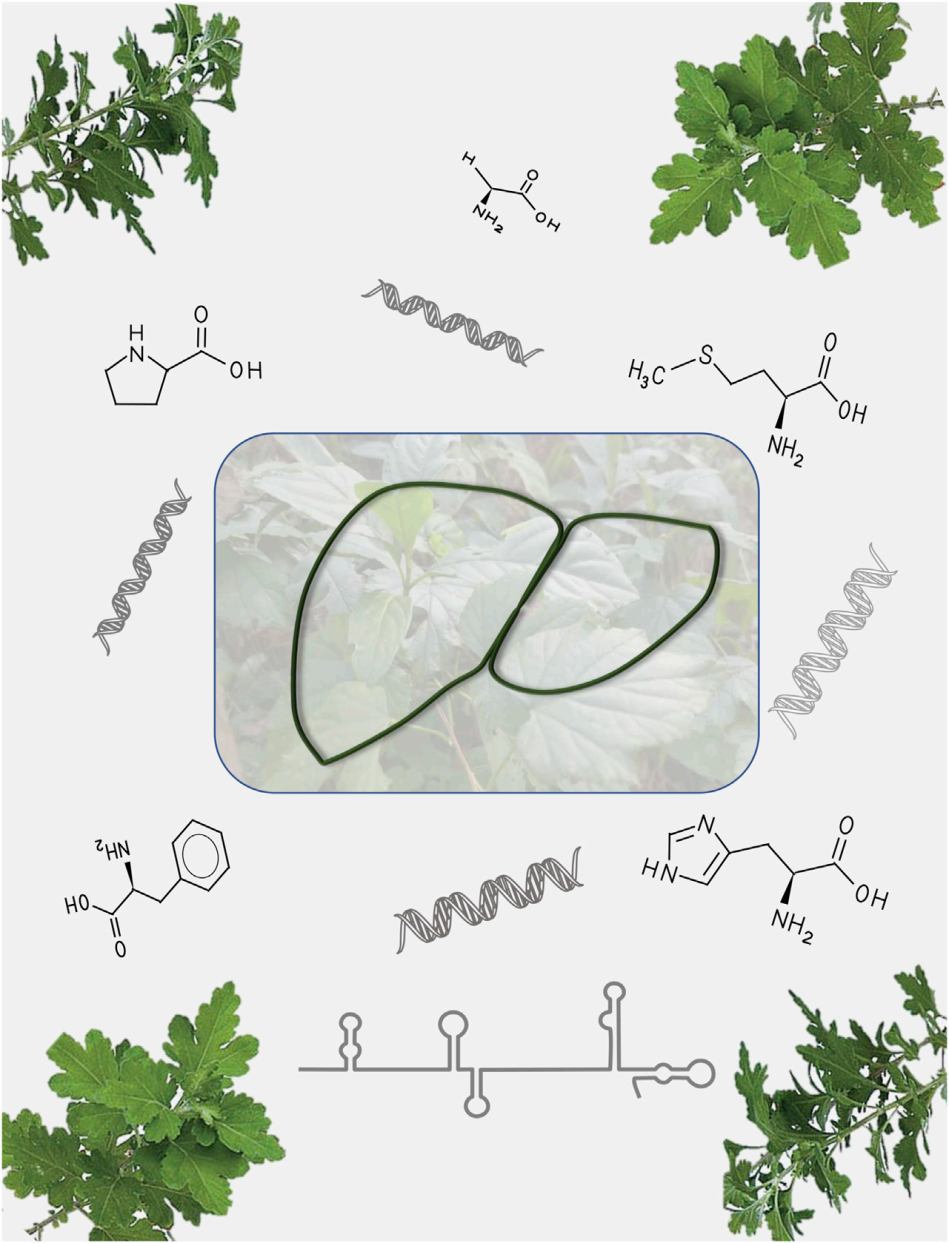
Nonalcoholic fatty liver disease: from pathogenesis to phytomedicine based on omics technology.

All these 4 articles show that multiomics research can contribute to the deep acknowledge of the pathogenic mechanisms of NAFLD as well as the pharmacological effects of phytomedicines (Figure 1).

